# Comprehensive Molecular Analysis of NSCLC; Clinicopathological Associations

**DOI:** 10.1371/journal.pone.0133859

**Published:** 2015-07-24

**Authors:** Ilenia Chatziandreou, Panagiota Tsioli, Stratigoula Sakellariou, Ioanna Mourkioti, Ioanna Giannopoulou, Georgia Levidou, Penelope Korkolopoulou, Efstratios Patsouris, Angelica A. Saetta

**Affiliations:** First Department of Pathology, Laikon General Hospital, Athens University Medical School, Athens, Greece; Yokohama City University School of Medicine, JAPAN

## Abstract

**Background:**

Selection of NSCLC patients for targeted therapy is currently based upon the presence of sensitizing mutations in EGFR and EML4/ALK translocations. The heterogeneity of molecular alterations in lung cancer has led to the ongoing discovery of potential biomarkers and targets in order to improve survival.

**Aim:**

This study aimed to detect alterations in EGFR, KRAS, BRAF, PIK3CA, MET-gene copy number and ALK rearrangements in a large cohort of 956 NSCLC patients of Hellenic origin using highly sensitive techniques and correlations with clinicopathological characteristics.

**Results:**

Mutations were detected in EGFR 10.6% (101 out of 956 samples), KRAS 26.5% (191 out of 720 samples), BRAF 2.5% (12 out of 471 samples), PIK3CA 3.8% (7 out of 184 samples), MET gene amplification was detected in 18% (31 out of 170) and ALK rearrangements in 3.7% (4 out of 107 samples). EGFR mutations were detected in exon 19 (61.4% of mutant cases), exon 21 p.Leu858Arg (19.8%), exon 20 (15.8%), exon 18 (2.9%) and were correlated with gender histology, smoking status and TTF1 staining. p.Thr790Met mutant cases (3.9%) displayed concurrent mutations in exons 19 or 21. Negative TTF-1 staining showed strong negative predictive value for the presence of EGFR mutations. KRAS mutations were associated with histology, the most common mutation being p.Gly12Cys (38%).

**Discussion:**

In conclusion, only 89 patients were eligible for EGFR -TKIs and ALK inhibitors therapy, whereas 257 patients showed other alterations, highlighting the necessity for a detailed molecular profiling potentially leading to more efficient individualized therapies for NSCLC patients.

## Introduction

Lung cancer remains the leading cause of cancer related mortality worldwide. Non- Small cell lung cancer (NSCLC) histology including adenocarcinoma, squamous cell carcinoma, large cell carcinoma, and bronchioloalveolar carcinoma, accounts for approximately 85% of all lung cancers [1, 2]. NSCLC patients have a poor prognosis, often diagnosed at an advanced stage due to the fact that early disease is typically asymptomatic. The overall 5- year survival has improved over the years but still remains at approximately 16–18% [3–5, 6] despite therapeutic advances.

Epidermal growth factor receptor (EGFR) is a transmembrane glycoprotein activates downstream RAS/RAF/MAPK, and PI3K/AKT signaling pathways, which cooperate to modulate several important mechanisms such as cell proliferation, adhesion, angiogenesis, migration, and survival [7]. Aberrant activation of EGFR could be triggered by mutation or amplification/ over-expression causing upregulation of oncogenic cell signaling and malignant transformation [8]. Activating mutations of EGFR kinase domain clustered in exons 18–21 are well established as predictive biomarkers for treatment of patients with EGFR tyrosine kinase inhibitors (TKIs) [9]. Lung cancer patients harboring such alterations show a 70% to 80% response rate to TKIs [10–12].

Although EGFR mutations are being used as either positive or negative predictive factors, accumulating data suggest a possible predictive value for alterations in other genes (KRAS, BRAF, PIK3CA, etc) which also affect the two major signaling pathways downstream of EGFR. In order to apply an individualized approach for a more efficient treatment of lung cancer patients, a molecular characterization is now mandatory, as part of baseline diagnostic procedures.

KRAS is a well-established predictive biomarker for colorectal cancer also implicated in lung carcinogenesis. KRAS mutations are found frequently in white patients with lung adenocarcinoma and smoking history [11, 13–16] and have been associated with poor prognosis and resistance to TKIs towards EGFR [17,18].

BRAF mutations, although detected at lower frequencies in lung cancer, have emerged as an alternative important mechanism of MAPK signaling activation downstream of KRAS. To date, BRAF has been successfully utilised as a therapeutic target in melanomas. The predictive value of BRAF mutations in NSCLC has not been clarified yet, although clinical trials with BRAF and MEK inhibitors in the NSCLC setting are ongoing in order to evaluate the clinical value of this potential biomarker [18–21].

PIK3CA gene encodes for the catalytic subunit of lipid kinase PI3K involved in signaling downstream of EGFR. Mutations in a broad spectrum of tumors, such as breast, bladder, colon, gastric cancer and glioblastomas [22, 23] and at much lower frequency in NSCLC cause aberrant activation of phosphatidylinositol 3-kinase (PI3K)/AKT signaling. Such alterations are considered as potentially useful biomarkers of resistance to EGFR-targeted therapy undergoing clinical validation.

MET gene, on chromosome 7q31, encodes a transmembrane tyrosine kinase receptor for HGF/scatter factor. Aberrant MET activation may be derived from overexpression, gene amplification or gene mutations. In NSCLC it has been linked with acquired resistance to EGFR TKIs. Therefore several MET inhibitors are being developed and tested as potential therapeutic strategies for NSCLC.

The *ALK* (anaplastic lymphoma kinase) gene rearrangement was originally identified in the context of a subtype of Non-Hodgkin lymphoma where *ALK* was fused to nucleophosmin (NPM) as a result of a chromosomal translocation [24]. In 2007, Soda et al [25] found the fusion of ALK gene with Echinoderm Microtubule-associated protein-like 4(EML4), as a consequence of a small inversion within chromosome 2p, in NSCLC tumors. This rearrangement leads to the production of a chimeric protein with constitutive ALK kinase activity, which promotes malignant growth and proliferation [26]. The incidence of EML4-ALK rearrangement ranges from 3–7% in NSCLC, depending upon the population studied and the detection methods used [27]. ALK rearrangements are more frequently observed in younger patients, light or never-smokers, adenocarcinoma histology with frequent signet ring cells [28, 29]. ALK rearrangement status is a critical biomarker to predict response to tyrosine kinase inhibitors, such as crizotinib which has been associated with significant reduction of tumor burden [30].

Taking into consideration the reported differences of mutation frequencies among various populations and ethnic groups, we aimed to examine the molecular profile of a large cohort of Greek NSCLC cancer patients, by performing mutational analysis in genes implicated in EGFR/RAS/MAPK, PI3K/AKT signaling and to search for relevant clinicopathological associations.

## Materials and Methods

### Ethics Statement

The present study was approved by the Laikon General Hospital University of Athens Ethics Committee (Protocol No. ΕΣ283/26-5-10). Since this was a retrospective study the Ethics Committee waived the need for an informed consent, and a policy of strict anonymity and confidentiality was assured. All patient data were anonymized and de-identified in a confidential manner. All information included in the data set was used exclusively for the purpose of this study, and was not shared with other individuals or organizations. Cases were randomly selected based on the availability of archival material from the database maintained in the First Department of Pathology, Laikon General Hospital, University of Athens Medical School, Greece.

### Patients

This is a retrospective study of 956 cases of non-small cell lung cancer (males = 681, females = 275 median age = 65; range years 29–94) for which archival material from primary tumours was available in our institute, a major pathology centre in Greece receiving samples from hospitals across the country. Samples were classified as Adenocarcinomas (n = 716), Squamous cell carcinomas (n = 87), Adenosquamous (n = 11), NSCLC NOS (n = 110), Large cell carcinomas (n = 5), Large cell Neuroendocrine carcinomas (n = 18), Pleomorphic and other carcinomas (n = 9), according to the International Association for the Study of Lung Cancer/American Thoracic Society/European Respiratory Society International Multidisciplinary Classification of Lung Adenocarcinoma/2011, WHO 4^th^ edition 2015 (Tables 1 and 2). This cohort included predominantly bronchoscopic biopsies and surgical specimens, but also a small number of bone marrow biopsies and cytology specimens. Complete information regarding tumour staging was available for 291 samples, due to the analysis of a large number of incisional biopsies. The distribution of tumour stages for 291 samples according the 7^th^ edition of lung cancer staging was as follows: 11 cases stage IA, 23 cases stage IB, 41 cases stage IIA, 24 cases stages IIB, 51 cases stage IIIA, 8 cases stage IIIB and 124 cases stage IV. Smoking status was available for 132 cases, current smokers or ex-smokers were defined as smokers whereas never smokers as non-smokers. TTF-1 staining data was available for 595 cases (Table 1).

**Table 1:** Clinicopathological and demographic characteristics of NSCLC patients.

Characteristics	Patients (n)	Characteristics	Patients (n)
All patients	956		
**Gender**		**Histology**	
*Female*	275	AdCa	716
*Male*	681	Squamous	87
**Age**		Adenosquamous	11
*≤50*	77	NSCLC NOS	110
*51–60*	181	Large cell carcinoma	5
*61–70*	279	Lage cell neuroendocrine carcinoma	18
*>70*	240	Pleomorphic carcinoma	8
*Median age*, *year (range)*	65 (29–94)	poorly differentiated NSCLC with spindle cell carcinoma	1
**Smoking**		**Stage**	
smokers	105	*IA*	11
*non-smokers*	27	*IB*	23
**TTF-1 staining**		*IIA*	41
*Positive*	419	*IIB*	24
*Negative*	176	*IIIA*	51
**Grade**		*IIIB*	8
*high-intermediate*	181	*IV*	124
*low*	325		

**Table 2:** Histological Classification of 956 NSCLC samples A. Classification of Resection Samples B. Classification of Small Biopsies/cytology samples and C. Metastasis Samples according to IASLC/ATS/ERS 2011, WHO 2015.

A. RESECTION SAMPLES	(n)	B. SMALL BIOPSIES/CYTOLOGY	(n)	SMALL BIOPSIES/CYTOLOGY	(n)
**Preinvasive Lessions**		**Adenocarcinoma**			
Adenocarcinoma in situ Non mucinous	4	with solid pattern	293	with acinar pattern and clear cell features	1
Adenocarcinoma in situ Mucinous	1	with acinar pattern	57	with solid or colloid pattern and signet ring features	7
**Invasive adenocarcinoma**		with papillary pattern	13	**Squamous**	
Lepidic predominant	9	with micropapillary pattern	1	squamous and NSCLC favor squamous	51
Acinar predominant	83	with solid and acinar pattern	21	**Other categories**	
Solid predominant	113	with solid or acinar and micropapillary pattern	6	NSCLC with squamous cell and adenocarcinoma patterns	10
Papillary predominant	18	with acinar and papillary pattern	3	NSCLC, NOS	65
Invasive mucinous adenocarcinoma	14	with lepidic pattern (an invasive component cannot be excluded)	10	NSCLC with neuroendocrine (NE) morphology, possible LCNEC	13
**Squamous cell carcinoma**		mucinous adenocarcinoma with lepidic pattern	1	poorly differentiated NSCLC with spindle cell carcinoma	1
Squamous	32	AdCa with colloid pattern	3	**Total**	**556**
Keratinizing squamous carcinoma	1				
**Other categories**					
Adenosquamous	2				
Combined large cell neuroendocrine carcinoma	2				
Large cell neuroendocrine carcinoma	2				
Large cell	5				
NSCLC NOS	39				
Pleomorphic	6				
**Total**	**331**				
**C. METASTASIS**	(n)				
**Adenocarcinoma**					
with solid pattern	45				
with acinar pattern	7				
with papillary pattern	1				
with solid and acinar pattern	4				
with solid or acinar and micropapillary pattern	1				
squamous	3				
Metastatic NOS	5				
NSCLC with neuroendocrine (NE) morphology, possible LCNEC	3				
**Total**	**69**				

### Genomic DNA isolation

#### DNA extraction from paraffin embedded tissues

Sections 10 μm thick were cut from—paraffin-embedded tissue blocks after macrodissection under the Light Microscope by an experienced pathologist for tumor enrichment. DNA was extracted from the selected tissue areas following a standard DNA extraction kit protocol (NucleoSpin tissue, Macherey–Nagel, Duren, Germany). The extracted DNA was quantitated on a Picodrop Microliter spectrophotometer. Laser Microdissection was applied in 68 cases on which macrodissection could not be performed due to low content of tumour cells, or vast dispersion of tumour cells, and high infiltration with normal or inflammatory cells. Microdissection was performed using a Leica LMD6000B laser capture system (Leica Microsystems CMS GmbH, Wetzlar, Germany). DNA was extracted using the Qiagen DNA Micro extraction kit.

### Molecular Analysis

#### High Resolution Melting Analysis (HRMA) and Cobas Method

KRAS, BRAF and PIK3CA mutations were screened using HRMA on a Light Cycler 480 (Roche Diagnostics, GmbH, Germany) in duplicate and further identified by Pyrosequencing and/or sequencing analysis. Each PCR reaction consisted of 20ng DNA, 0.3μM of each primer, 10μl LightCycler 480 HRM Master Mix (Roche Diagnostics, GmbH, Germany), 3.5mM MgCl_2_ in a total volume of 20μl. The thermal profile used in the Light Cycler was: 95°C for 10 min, followed by 50 cycles of 95°C for 10 sec, with annealing temperatures at 56°C–KRAS, 60°C–EGFR/BRAF and 62°C- PIK3CA for 15 sec, 72°C for 7 sec. The sequences of the primers for EGFR, BRAF, KRAS and PIK3CA have been published previously [24, 25]. EGFR mutations were examined using HRMA/pyrosequencing as described above or by the Cobas method (“Cobas EGFR test”, Roche Molecular Systems, Inc, Branchburg, NJ, USA) according to the manufacturer’s protocol. The Cobas EGFR test is designed to detect 41 mutations displaying maximum clinical significance in exons 18, 19, 20, and 21. Samples with limited material (biopsies) were selected for analysis with the cobas test, in order to minimize the DNA input requirements. In these cases an additional examination of exon 21 using HRMA was performed for p.Leu861Gln mutation detection.

#### Pyrosequencing/Sequencing

Alterations in KRAS exon 2, BRAF exon 15 and EGFR exons 18–21 observed by HRMΑ, were identified by Pyrosequencing using the Pyromark Gold Q24 Reagent kit with the Q24 Pyrosequencer (Qiagen GmbH, Hilden, Germany) according to the manufacturer’s protocol as previously described [31,32]. Sanger Sequencing was used to identify mutations in BRAF (except Val600Glu) and PIK3CA gene exons 9 and 20. Briefly PCR products positive by HRMA were sequenced using the BigDye terminator cycle sequencing kit (Applied Biosystems, CA, USA) in order to confirm the presence of mutations. The sequencing products were analysed on an ABI Prism 310 Genetic Analyzer (Applied Biosystems). PCR primers were also used for sequencing analysis. Results were verified by sequencing analysis of at least two independent PCR products. PIK3CA gene exon 9 sequence analysis did not show amplification of the pseudogene.

#### Relative copy number analysis

MET relative copy number was determined for 170 samples by Real Time PCR. GADPH was used as a reference gene. The primer sequences used for each gene were as follows: MET gene Forward *5’-ACGGTCCAAAGGGAAACTCT-3’*, Reverse 5’-*CTCCAGAGGCATTTCCATGT-3’*, GADPH gene Forward 5’-CAATTCCCCATCTCAGTCGT-3’, Reverse 5’- GCAGCAGGACACTAGGGAGT-3’. Results are expressed as normalized ratio calculated using a calibrator sample consisting of a pool of normal DNAs extracted from blood from healthy subjects. A standard curve from 5 consecutive serial dilutions of normal human genomic DNA was used to determine the reaction Efficiency (E). The data were analysed using the efficiency corrected calculation models, based on one sample [33]:

$$\text{Ratio}={\left(\text{E target}\right)}^{\Delta \text{Cp target}(\text{calibrator-sample})}÷{\left(\text{E ref}\right)}^{\Delta \text{Cp ref}(\text{calibrator-sample})}$$ (1)

The cut off value was established as previously described [34]. Briefly the mean of normalized ratio values of 20 normal lung DNA samples was calculated and a lung tumor sample was considered as amplified if its normalized ratio was over Mean+2SD. All specimens were analysed in duplicate.

#### Immunohistochemistry- IHC

IHC was applied on 4μm thick formalin-fixed paraffin-embedded tissue sections with the prediluted rabbit monoclonal IVD anti-ALK antibody D5F3 (Ventana Medical Systems, Tuscon, AZ) on the Ventana Benchmark XT system, using the Optiview DAB detection/ Amplification kit according to the manufacturer’s instructions. IHC was evaluated by two experienced pathologists, using the recommended scoring algorithm. IHC detects ALK rearrangements independently of the fusion partner and represents a reliable screening approach [35].

#### Fluorescent In Situ Hybridisation–FISH

FISH was used in order to verify positive IHC results according to recent guidelines [36, 37]. FISH was performed on 4 μm–thick paraffin slides from FFPE tumor tissues, using an established break-apart probe (IVD) specific to the ALK locus (Vysis ALK Break Apart FISH Probe Kit, Abbott Molecular, Abbott Park, Illinois, USA) according to the manufacturer’s instructions. Stained slides were evaluated by two trained pathologists under a 100x oil immersion objective with a fluorescence microscope using the scoring criteria defined by the manufacturer. Only tumor cells of which the nuclei had one or more FISH signals of each color were enumerated. A positive cell was defined as one displaying split signals (two or more signal diameters apart), or a single orange signal (deleted green signal) in addition to fused and/or split signals. A sample was considered positive if >25 cells out of 50 were positive. If a sample had 5 to 25 positive cells (10 to 50%) another 50 tumor cells were counted and the sample was considered positive if the average percentage of positive cells was >15% [37].

### Statistical analysis

Statistical analysis was performed in order to correlate mutational status with other parameters such as gender, histological type, grade and stage using Pearson’s Chi square and Fisher’s exact test where appropriate. Statistical calculations were performed using the statistical package SPSSv21.0 for Windows. All results with a two-sided p-value <0.05 were considered significant. Associations of mutational status with TNM stage were limited to samples with available clinical data.

## Results

### Patient characteristics

All patients were of Hellenic origin and the available clinicopathological characteristics are presented in Table 1. Median age was 65 years (range 29–94 years) and the analysed cohort exhibited a male predominance (71%), with a 2.4:1 male to female ratio. Regarding histological classification specimens were mainly adenocarcinomas at 75% followed by squamous cell carcinomas at 9%. There was statistical significant correlation between tumor stage and age of the patients (p = 0.01), in older patients ≥65 years old (75%), higher stage (III/IV) tumors were more common than earlier stage (I/II) tumors. The age of the patients was also associated with gender (p = 0.003), 63% of female patients were under 65 years of age and 49% of male patients were over 65 years of age. In the subset of patients with known smoking status, gender was related with smoking history as males were more frequently smokers (p<0.0001). Furthermore, it was observed that SCC histology was associated with gender (p = 0.002) as SCC tumors were more common in men (75 of 87 squamous cases) than women (12 of 87 squamous cases). There was also a marginal correlation of grade and stage of tumors (p = 0.054) where, well and moderately differentiated cases associated with stages I/II while low differentiated tumors with stages III/IV. TTF-1 staining was associated with adenocarcinoma histology (p<0.0001).

### EGFR Mutational analysis and correlation to clinico-pathological data

956 tumor samples from patients with NSCL cancer were tested for the presence of activating mutations in exons 18, 19, 20 and 21 of the EGFR gene. It is important to note that mutations were detected in several cases with low tumor or cell content, indicating the high sensitivity of the methods used. Mutational analysis was feasible even in cases with tumor content as low as 10% or low cellular content.

**Fig 1:**
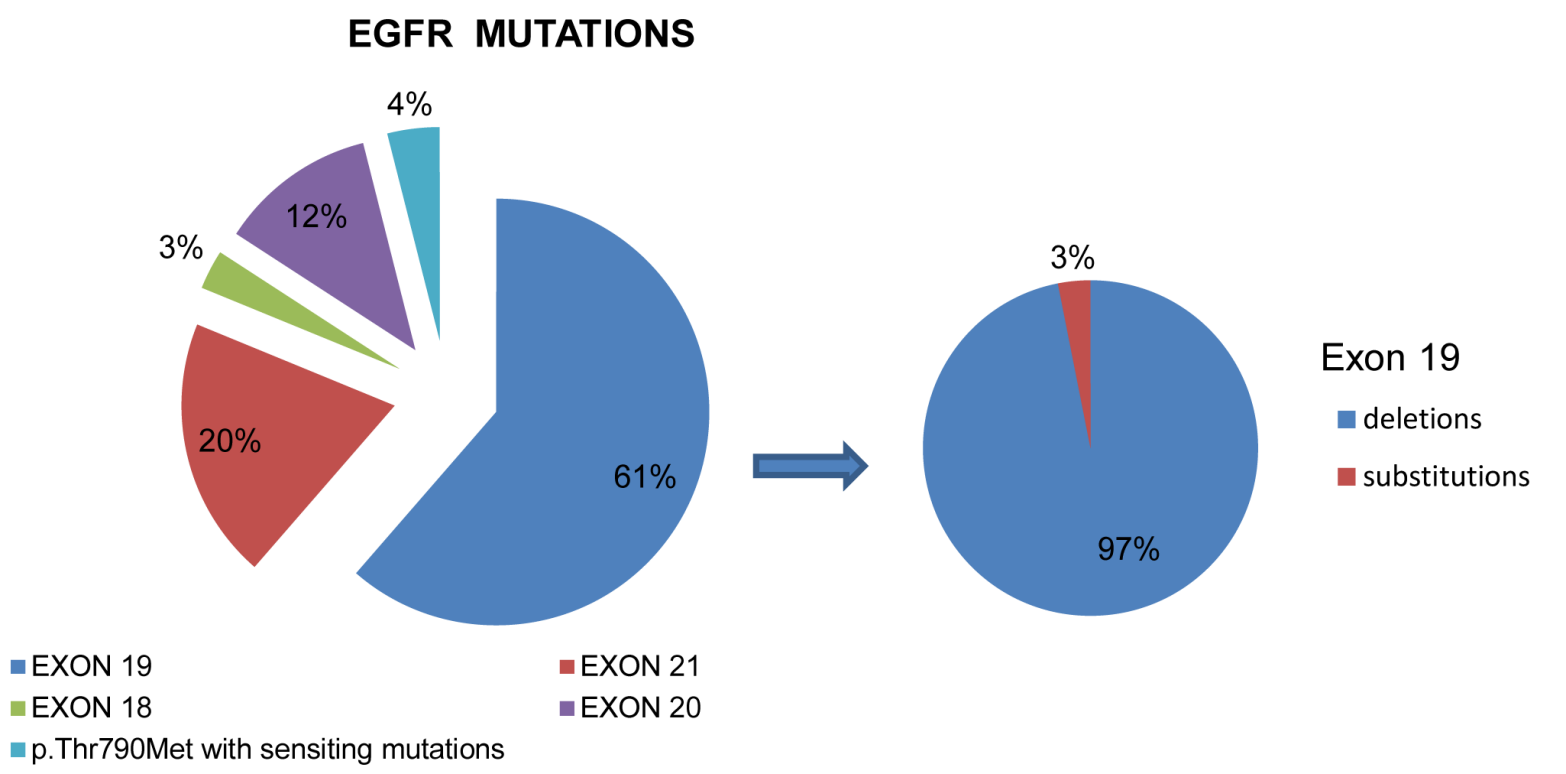
Frequency of mutations (%) in exons 18, 19, 20 and 21 of EGFR gene.

EGFR mutations were detected in 10.6% (101 out of 956 samples) of the examined cases (Table 3). Most identified mutations were sensitizing, namely deletions in exon 19 (61.4%, 62 cases out of 101, Fig 1) the majority of which were identified as deletion of 15 bases starting at nucleotides 2236 or 2235 leading to a deletion of Glu-Leu-Arg-Glu-Ala (p. Glu746_Ala750delELREA). The rest of exon 19 mutations were identified as: p.Leu747_Glu749delLRE, p.Leu747_Thr751delLREAT, p.Glu746_Thr751delELREAT, p.Lys745_Ala750delKELREA, p.Ile744Met and also a rare point mutation, p.Leu747Pro. Exon 21 was found mutated at lower frequency (21.7%, 22 out of 101 cases). A well-known G to T transversion at nucleotide 2573 leading to p.Leu858Arg amino acid substitution was seen in 20 cases. Furthermore, one sample displayed a mutation p.Gly863Val and another sample displayed two point mutations, namely p.Val834Leu along with p.Leu858Arg. In addition, sixteen samples (15.8%) were mutated in exon 20, the majority of which displayed insertions. The resistance mutation p.Thr790Met in exon 20 was found in 4 cases all of which showed also sensitizing mutations (3 cases in exon 19 and one in exon 21). Exon 18 mutations, namely p.Gly719Ala, were found in three samples (2.9%) in our cohort (Fig 1).

**Table 3:** Mutational analysis of NSCLC samples. This table shows the percentage of mutations in this cohort (All NSCLC) and the two major histological subtypes; adenocarcinomas (AdCa) and squamous cell carcinomas (Squamous).

	All NSCLC		AdCa		Squamous	
	(n)	(%)	(n)	(%)	(n)	(%)
**EGFR**						
*Wild type*	855	89.4	624	87.2	87	100
*Mutant*	101	10.6	92	12.8	0	0
**KRAS**						
*Wild type*	529	73.5	385	70.1	64	97
*Mutant*	191	26.5	164	29.9	2	3
**BRAF**						
*Wild type*	459	97.5	369	97.4	37	95
*Mutant*	12	2.5	10	2.6	2	5
**PIK3CA**						
*Wild type*	177	96.7	139	96.5	25	96.2
*Mutant*	7	3.8	5	3.5	1	3.8
**MET Amplification**						
*Absent*	139	82	85	85	34	72.4
*Present*	31	18	15	15	13	27.6
**ALK rearrangement**						
*Absent*	103	96.3	72	94.7	12	100
*Present*	4	3.7	4	5.3	0	0

**Fig 2:**
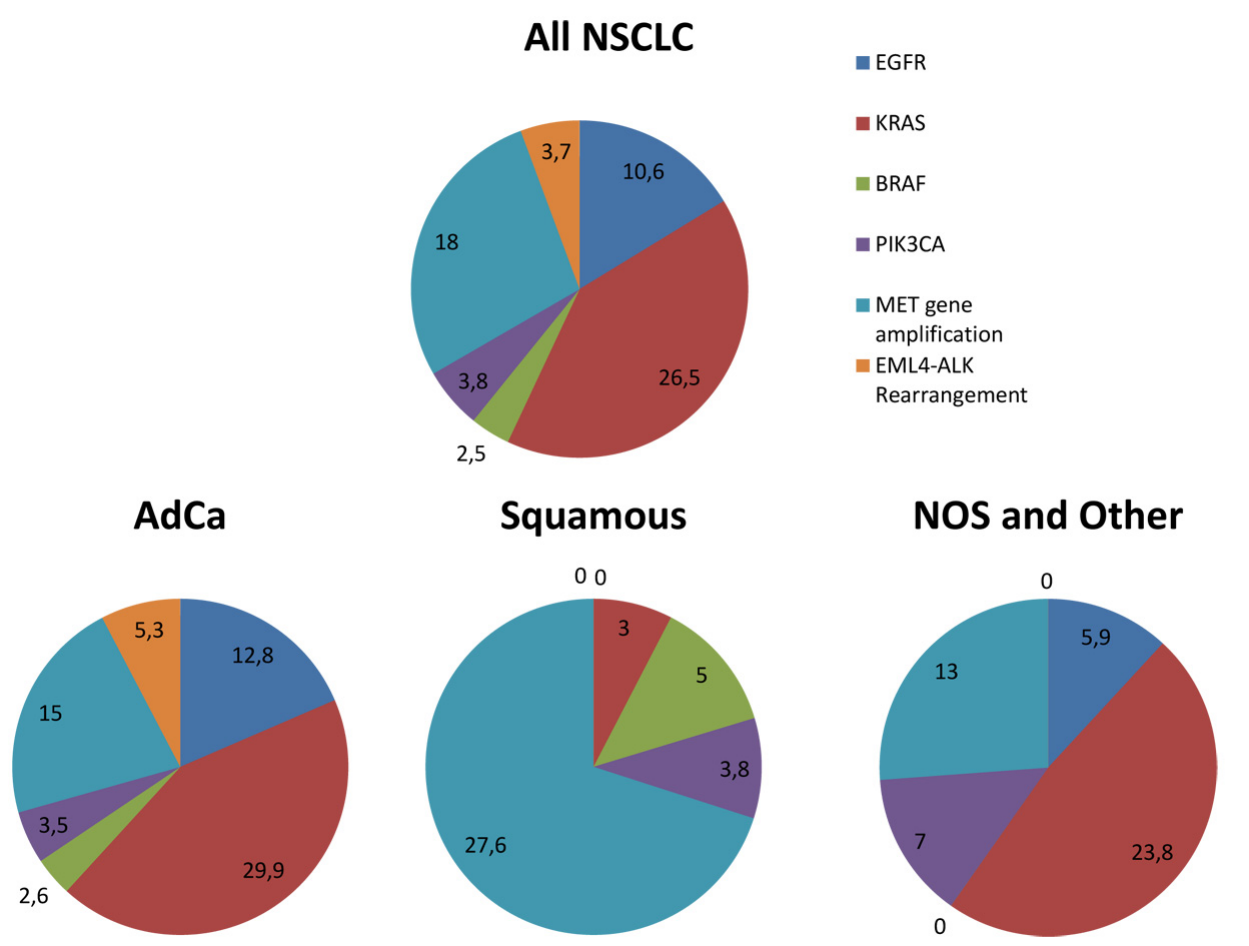
Pie charts representing the frequencies (%) of alterations of the examined genes in this cohort. Percentage of alterations for all the samples of the cohort (All NSCLC) and between the different histological types adenocarcinoma (AdCa), squamous cell carcinomas (Squamous), and other types (NOS and Other).

The percentage of mutated adenocarcinomas was 12.8% (92 out of 716 adenocarcinomas), whilst in all other histological types the percentage of EGFR mutations did not surpass 5% (9 out of 240 samples), and no mutation was detected in squamous cell carcinomas (Fig 2, Table 3). As far as adenocarcinoma groups are concerned, EGFR mutations were more frequent in lepidic (4 out of 19 samples, 21%), micropapillary (either pure or mixed) (2 out of 6 samples 33%), non-mucinous in situ AdCa (2 out of 4 samples 50%), while in all other groups the percentage ranged from 8 to 16% (Table 4), but no statistical correlations were elicited. The presence of EGFR mutations correlated with adenocarcinoma histology (p<0.001), with gender (p<0.001) as it was encountered at higher rates in females (21.3%) than in males (7%), and were also associated with smoking status (p<0.001), in particular EGFR mutations were present in 8 of 105 (7.6%) smokers and 12 of 27 (44%) non-smokers. Additionally, EGFR mutations were mutually exclusive with KRAS mutations (p<0.001). Statistical significant correlations were not found with other clinicopathological characteristics such as age (p = 0.137), grade (p = 0.587) or stage (p = 0.097). It has to be noted that cases with advanced stage (20 of 183 cases with Stage III/IV) were found more frequently mutated (11%) than those with early stage (5%) (5 of 99 cases with Stage I/II) but the association was not statistically significant.

**Table 4:** EGFR mutations distributed amongst different Adenocarcinoma groups.

	EGFR		
	NL	MT	(%)
In situ Non mucinous AdCa	2	2	50
lepidic	15	4	21
acinar	124	23	15.6
solid	399	52	11.5
papillary	26	5	16.1
Invasive mucinous AdCa	12	2	14.3
More than one growth pattern present	22	2	8.3
AdCa with micropapillary pattern (either pure or mixed)	4	2	33

Of the cases with available TTF-1 data, only 2 of 74 EGFR mutant cases were TTF1-negative therefore EGFR mutations were also associated (p<0.0001) with TTF-1 positivity (Negative predictive value, 98.8%).

### Alterations in KRAS, BRAF, PIK3CA and correlations to clinicopathological data

**Fig 3:**
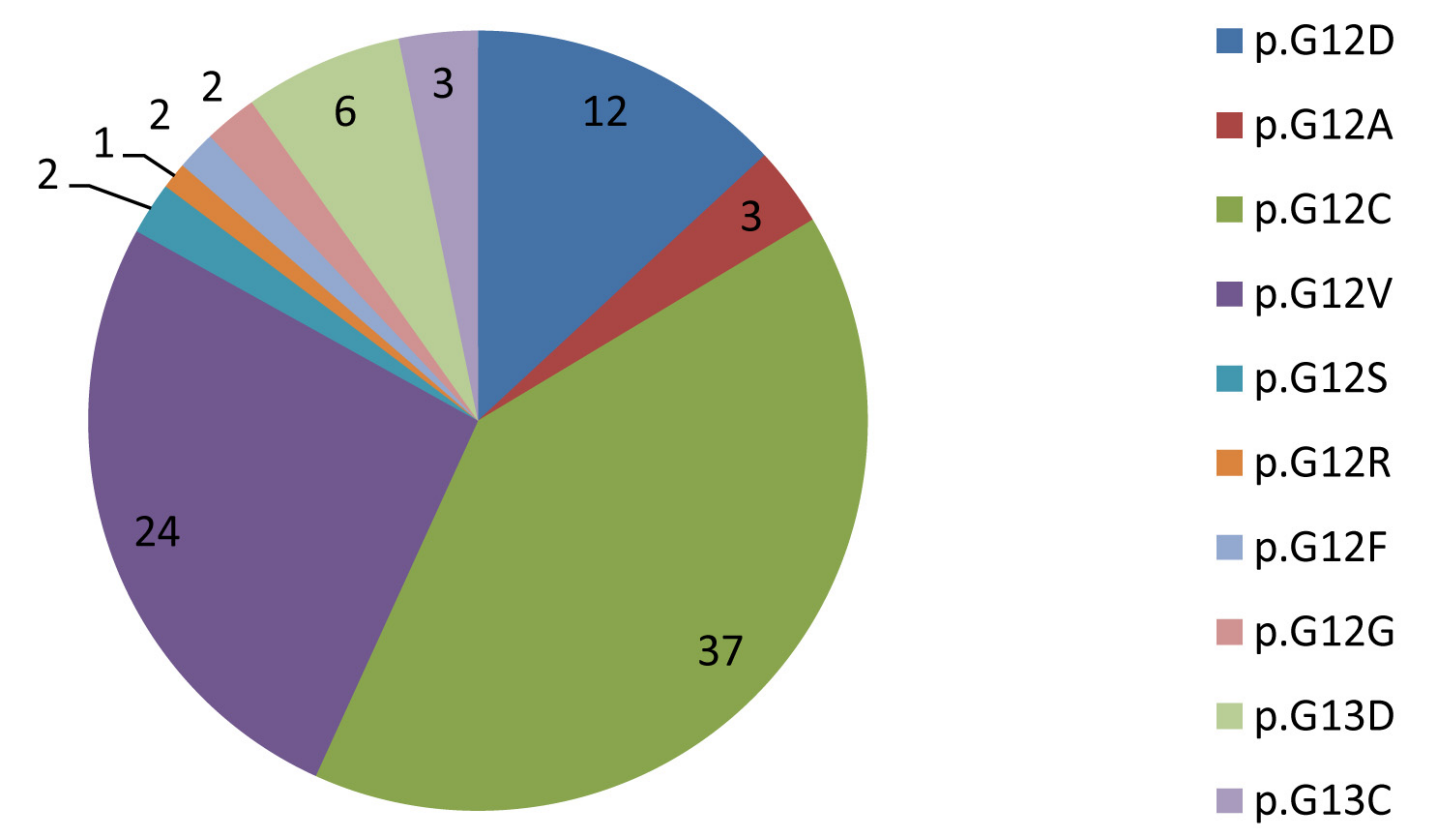
Schematic representation of the distribution (%) of different KRAS mutations.

In order to further examine the mutational profile of the patients, as far as MAPK and PI3K/AKT signaling pathways are concerned, HRM analysis and Pyrosequencing/sanger sequencing were applied to detect mutations in KRAS (exon 2), BRAF (exon 15) and PIK3CA (exons 9 and 20) genes. KRAS mutational analysis was performed for 720 samples and 26.5% (191 out of 720) of the cases were mutated, mainly adenocarcinomas (29.9%, 164 out of 549 adenocarcinomas). Regarding the adenocarcinoma groups the presence of KRAS mutations was more common in Invasive mucinous AdCa (5 out of 11 samples, Table 5), but no statistical correlations were elicited. In the other NSCLC histological types KRAS mutation frequency was 3% in squamous cell carcinomas (2 out of 66, Table 3) and 19% in NOS and other histologies (25 out of 105 Fig 2). Interestingly, a statistically significant relation (p<0.0001) depicting the association of KRAS mutations with tumors’ histological type in NSCLC patients was determined. In contrast with EGFR, KRAS mutations were equally distributed between the two sexes, observed in 26.4% of males and 25.9% of females. KRAS mutations mainly affected codon 12 (92%) and the distribution of mutations was the following: the most frequent mutation was p.Gly12Cys (38% of mutant cases) which has been reported to be smoking related and p.Gly12Val (24%). In detail, ten different mutations were identified in total namely: p.Gly12Cys, p.Gly12Val, p.Gly12A, p.Gly12Asp, p.Gly12Arg. p.Gly12Ser, p.Gly12Phe, p.Gly12Gly, p.Gly13Asp and p.Gly13Cys (Fig 3). From the cases analysed for KRAS, smoking status was available for only 84 patients. KRAS was mutated in 21 of 70 smokers (30%) and 1 of 14 (7.1%) non- smokers; although 21 out of 22 mutations were found in smokers, this finding was not statistical significant (p = 0.1), probably due to the small number of cases and in particular the limited number of non-smoking patients (14 patients). No clinicopathological associations were found between KRAS mutations and stage (p = 0.586), grade (p = 0.582) or age (p = 0.294).

**Table 5:** KRAS mutations distributed amongst different Adenocarcinoma groups.

	KRAS		
	NL	MT	(%)
In situ Non mucinous AdCa	1	1	50
lepidic	12	3	20
acinar	80	34	29.8
solid	248	102	29.1
papillary	16	8	33.3
Invasive mucinous AdCa	6	5	45.5
More than one growth pattern present	11	7	38.9
AdCa with micropapillary pattern (either pure or mixed)	5	1	16.7
Colloid	5	2	28.6

BRAF mutational screening was performed for 471 samples. Analysis of exon 15 (activation segment) revealed mutations in 2.5% of the cases (12 out of 471) mainly adenocarcinomas of different groups as shown in Table 6 (10 out of 12 mutant samples) and only two squamous cell carcinomas were found mutated (Fig 2, Table 3). Mutations were identified as T to A transition at nucleotide 1799 leading to a Valine to Glutamine substitution at codon 600, (p.Val600Glu) in 8 cases, and 4 cases exhibited non- p.Val600Glu, namely p.Ser614Ser, p.Thr589Ala, p.Ala598Val, and pVal600>TyrMet. BRAF mutations were not correlated with clinicopathological data such as histological type (p = 0.667), stage (p = 1.0), grade (p = 1.0), age (p = 0.310) or gender (p = 0.762).

**Table 6:** Frequencies of alterations between different groups of adenocarcinomas (NL = Normal, MT = Mutant, AMPL = gene amplification).

	BRAF				PIK				MET		
AdCa	NL	MT	(%)	AdCa	NL	MT	(%)	Adca	NL	AMPL	(%)
acinar	28	1	3.4	acinar	11	1	8.3	acinar	16	3	15.8
solid	170	7	3.9	solid	45	4	8.2	solid	48	9	15.8
AdCa with micropapillary pattern (either pure or mixed)	2	1	33.3					More than one pattern	4	1	20
colloid	2	1	33.3					lepidic	2	1	33.3
								colloid	0	1	100

As far as PIK3CA gene alterations are concerned, 184 samples were analysed for PIK3CA mutations in both exons 9 and 20, showing a mutation frequency of 3.8% (7 out of 184) (Fig 2, Tables 3 and 6). The mutations detected were mainly clustered in exon 20. The only PI3KCA exon 9 mutation, namely p.Glu545Lys, was present in a squamous cell carcinoma. Interestingly, most of the PIK3CA mutations in exon 20 coexisted with other mutations. More specifically they were found simultaneously with EGFR (p.Lys745_Ala750 del KELREA), KRAS (p.Gly13Cys, p.Gly12Val) or BRAF (p.Val600Glu) mutations. PIK3CA mutations were not associated with any of the clinicopathological characteristics in this cohort.

### MET gene copy number amplification and associations with clinicopathological features

MET gene was found amplified in 18% (31 out of 170 cases)(Fig 2, Tables 3 and 6). More specifically *MET* amplification was found in 15 of 100 (15%) ADC and 13 of 47 (27.6%) SCC; 8 of 38 (21%) women and 23 of 132 (17.4%) men. *MET* amplification was detected at similar frequencies in younger (under 65 years) and older ages (over 65 years), 19% and 17% respectively. Regarding tumor grade, MET amplification was observed in 18% of well/moderately differentiated tumors (7 of 39) and 19.3% (11 of 57) in low differentiated tumors. MET was found amplified in both EGFR wild type and mutant samples at similar percentages 18% (29 of 158) and 17% (2 of 12) respectively. In detail, one MET amplified sample displayed a double EGFR mutation, namely a deletion in exon 19 and p.Thr790Met in exon 20 and the other sample exhibited an insertion in exon 20. In addition, MET amplification was more common in KRAS wild type than KRAS mutant samples as it was detected in 16 of 84 (19%) and in 2 of 26 (7.7%) of KRAS wild type and mutant samples respectively. MET gene amplification was not correlated with any clinicopathological features.

### ALK rearrangement status

Immunohistochemical analysis was performed on 107 cases in order to evaluate ALK status. 3.7% of the samples (4 out of 107) were identified as ALK positive by IHC and validated by FISH. More specifically *ALK* rearrangement was found in 4 adenocarcinomas (1 female, 3 male patients) all with advanced stage disease. ALK rearrangement was mutually exclusive with the other examined alterations.

## Discussion

The characterization of EGFR activating mutations which predict sensitivity or resistance to anti-EGFR therapies has provided a basis for selecting lung cancer patients for targeted therapies. Currently in clinical practice patients with specific *EGFR* mutations or echinoderm microtubule-associated protein-like 4/anaplastic lymphoma kinase *(EML4/ ALK)* fusion gene could be treated with EGFR or ALK tyrosine kinase inhibitors (TKIs) respectively. However these biomarkers are clinically relevant for a limited subset of patients, approximately 15% in populations of European descent, and consequently the search for additional predictive biomarkers is ongoing in NSCLC.

In the present study we illustrate the mutation spectrum of NSCLC patients of Hellenic origin. NSCLC samples were examined following either macro or Laser micro dissection for the presence of driver mutations in EGFR (n = 956), KRAS (n = 720), BRAF (n = 472), PIK3CA (n = 184) genes, MET gene copy number amplification (n = 170), ALK gene rearrangement (n = 107) in an effort to define the occurrence of molecular alterations in NSCLC and their possible contribution to clinical decision making. The median age of NSCLC in our cohort (65 years) is similar to the median age worldwide (70 years) [6]. We report 85 sensitizing EGFR mutations among which four coexisted with resistant mutation p.Thr790Met, and 12 non sensitizing mutations in exon 20. In our study 10.6% of the examined NSCLC samples displayed EGFR mutations and the respective frequency reached 12.8% in adenocarcinomas in accordance with current results on populations of European descent [38–42]. There is a remarkable ethnical variation of EGFR mutation frequency, ranging from 9 to 12% in European populations [11, 39, 40, 42, 43] and reaching 40% in populations of South East Asia [9, 44–46]. It has been reported that more than 80% of the detected mutations are deletions clustered between codons 746 and 753 in exon 19 and point mutations affecting codon 858 in exon 21 [11, 47, 48]. Similarly, exon 19 alterations detected in this cohort accounted for 60% of the mutations identified with the most frequent being deletion p.Glu746-Ala750del. The second most common mutation detected (20%) was p.Leu858Arg in exon 21, followed by insertions in exon 20 (12%). Only three samples were mutants in exon 18 (p.Gly719Ala). Exon 20 point mutation p.Thr790Met was present at a frequency of 4% of mutant cases. Mutations were statistically correlated with adenocarcinoma histological type, non-smoking patients, female gender and TTF-1 positive staining, findings which are in line with the existing literature. TTF-1 staining showed a correlation with EGFR mutation status, displaying a high negative predictive value of 98.8% reinforcing previous data that bring out TTF-1 IHC as a clinical feature that may reliably estimate the absence of mutations with a great potential for the clinician [41, 42, 49].

KRAS mutations were the most common alteration detected in our cohort accounting for 26.5% of the examined cases and correlated with adenocarcinoma histology. In total, ten different alterations were identified the smoking related mutation p.Gly12Cys at codon 12 represented 38% of all KRAS alterations, followed by p.Gly12Val (24%), in accordance with results from previous studies [50, 51]. Interestingly, p.Gly12Asp which is the most common mutation in colon cancer adenocarcinomas was present in 12% of the mutant cases in our NSCLC cohort. The significance of KRAS mutations as a biomarker for NSCLC, remains elusive and although it has been linked with resistance to anti-EGFR therapy, recent bibliography suggests a possible clinical significance amongst the various KRAS mutations [50, 52]. It has been hypothesized that due to non-identical biological activities of different RAS alleles, different signaling outputs could be induced subsequently leading to variation of sensitivity to drugs. Recently a MEK inhibitor, selumetinib, in combination with docetaxel has been used in patients with KRAS-mutant tumors with promising efficacy [52–54].

Analyzing downstream the MAPK signaling pathway, at the level of B-RAF gene, low prevalence of BRAF mutations was encountered (2.5% of the cases) in our cohort in accordance with recent investigations in which 2–5% incidence is reported [20, 55, 56]. Albeit the low incidence of BRAF mutations, it has been proposed that NSCLC patients carrying Val600Glu mutation may potentially benefit from treatment with selective inhibitors, currently in clinical trials, highlighting the importance of prospective genotyping of NSCLC patients for BRAF mutations. BRAF mutations in this cohort were not confined to samples displaying adenocarcinoma histology but were also found in two SCC cases. In addition, a high percentage (33%) of the mutant cases exhibited non-pVal600Glu mutations, observations which are in line with recent studies [55–57]. BRAF mutation has been related with smoking status in recent reports [55–57], an analysis not feasible in this cohort due to the lack of data for smoking status for BRAF mutant cases. The detected frequency of Val600Glu mutation is in agreement with previous studies of populations of European descent [55–57], but it differs from the results of a cohort of 5125 Chinese NSCLC patients, in which it was present in only 0.5% of the cases, associated with gender, but not with smoking status [58].

As far as PIK3CA gene mutations are concerned we detected a low frequency of mutations (3.3%) by analyzing a subset of 184 NSCLC samples, which is in accordance with previous studies [51, 59–61]. Interestingly, similar rates of PIK3CA point mutations have been observed between East Asian [44, 62] and patients of European descent [22, 51, 61] although a difference on the type of mutations identified is noted; there seems to be a higher incidence of PIK3CA exon 9 mutations in East Asian patients [34, 60, 62, 63]. In this cohort, PIK3CA point mutations coexisted with mutations in other examined genes such as EGFR, KRAS and BRAF and no significant correlation with clinicopathological factors was elicited, in line with current literature [43–49]. An association of PIK3CA mutations with TKI resistance and poor survival in NSCLC patients treated with EGFR-TKIs has been reported [61]. Recently, it has been suggested that patients presenting with advanced cancer showing p.His1047Arg mutation (exon 20) may be more sensitive to PI3K/AKT/mTOR pathway inhibitors [64], although preclinical and early clinical studies imply that KRAS and PIK3CA concurrent mutations may induce resistance to such inhibitors [64, 65].

MET relative copy number variation analysis by qRT-PCR showed gene amplification in 18% of the NSCLC cases examined. This percentage is in accordance with similar investigations in which MET gene copy amplification in TKI-chemo naïve patients ranges from 5–20% [27, 66–70], although higher incidence (45%) has been reported in one study [66]. This variability could be attributed to different methods of analysis and cut off values as well as to population differences. Although MET amplification has been linked to acquired resistance to TKI treatment, the present study as well as previous investigations on chemo-TKI naïve patients, are in favor of a pre-existing nature of this alteration. MET amplification has been associated with poor prognosis, increased proliferation, tumor invasiveness and angiogenesis [71, 72]. Conflicting data regarding clinicopathological associations of MET alterations with gender, smoking and histology exist [67, 70, 72]. In the present study no clinicopathological associations with MET amplification were defined, in accordance with other reports [56, 68].

Regarding ALK rearrangement, in our study ALK positive cases analysed by IHC and confirmed by FISH were found in 3.7% of the cases. This frequency is consistent with previous reports demonstrating ALK positivity at a range of 3.0% to 7.0% [25,30,73]. No statistically significant associations with the clinicopathological parameters were found which is in accordance with previous studies [74]. Recently, two large meta-analysis studies have elicited correlations with never/light smoking history, female gender, adenocarcinoma histology, as well as non-Asian patients with advanced stage [40,75]. It is worth noting that in our study although no significant statistical correlations were found, possibly due to the limited number of cases, the positive samples were advanced stage adenocarcinomas.

To conclude, in this study we report the frequency of EGFR mutations in a cohort of 956 lung cancer patients as well as the frequency of alterations in KRAS, BRAF, MET, PIK3CA and ALK genes in a subset of these patients. The selection of NSCLC patients for targeted therapies to date is exclusively based on EGFR and ALK mutational status, accounting for a small percentage of patients. Accordingly, in this cohort only 89 patients were eligible for therapy with EGFR TKIs or ALK inhibitors. On the other hand, a subset of 207 patients displayed non sensitizing EGFR and KRAS mutations, and further 50 patients showed alterations in BRAF, PIK3CA or MET genes. New targeted drugs currently in development and clinical trials would increase the number of patients eligible for targeted therapeutic approaches that may overcome acquired resistance to TKIs. Our findings in this large cohort of NSCLC patients, add to a growing body of data concerning NSCLC molecular profiling, highlighting the necessity of a more detailed molecular analysis potentially leading to more efficient individualized therapies.
